# The Role of Executive Functions in the Development of Empathy and Its Association with Externalizing Behaviors in Children with Neurodevelopmental Disorders and Other Psychiatric Comorbidities

**DOI:** 10.3390/brainsci10080489

**Published:** 2020-07-28

**Authors:** Chiara Cristofani, Gianluca Sesso, Paola Cristofani, Pamela Fantozzi, Emanuela Inguaggiato, Pietro Muratori, Antonio Narzisi, Chiara Pfanner, Simone Pisano, Lisa Polidori, Laura Ruglioni, Elena Valente, Gabriele Masi, Annarita Milone

**Affiliations:** 1 IRCCS Stella Maris Foundation, 56128 Pisa (Calambrone), Italy; ccristofani@fsm.unipi.it (C.C.); gsesso@fsm.unipi.it (G.S.); pcristofani@fsm.unipi.it (P.C.); pfantozzi@fsm.unipi.it (P.F.); einguaggiato@fsm.unipi.it (E.I.); pmuratori@fsm.unipi.it (P.M.); anarzisi@fsm.unipi.it (A.N.); cpfanner@fsm.unipi.it (C.P.); lpolidori@fsm.unipi.it (L.P.); lruglioni@fsm.unipi.it (L.R.); evalente@fsm.unipi.it (E.V.); gmasi@fsm.unipi.it (G.M.); 2 Department of Clinical and Experimental Medicine, University of Pisa, 56126 Pisa, Italy; 3 Department of Neuroscience, AORN Santobono-Pausilipon, 80122 Naples, Italy; pisano.simone@gmail.com; 4 Department of Translational Medical Sciences, Federico II University, 80138 Naples, Italy

**Keywords:** empathy, executive functions, attention deficit and hyperactivity disorder, autism spectrum disorder, disruptive behavior disorders

## Abstract

Executive functions have been previously shown to correlate with empathic attitudes and prosocial behaviors. People with higher levels of executive functions, as a whole, may better regulate their emotions and reduce perceived distress during the empathetic processes. Our goal was to explore the relationship between empathy and executive functioning in a sample of children and adolescents diagnosed with Attention Deficit and Hyperactivity Disorder alone or associated with comorbid Disruptive Behavior Disorders and/or Autism Spectrum Disorder. We also aimed to examine the role of empathic dimensions and executive skills in regulating externalizing behaviors. The 151 participants with ADHD were assigned to four groups according to their psychiatric comorbidity (either “pure” or with ASD and/or ODD/CD) and assessed by means of either parent- or self-reported questionnaires, namely the BRIEF−2, the BES, and the IRI. No questionnaire was found to discriminate between the four groups. Affective Empathy was found to positively correlate with Emotional and Behavioral Regulation competences. Furthermore, Aggressiveness and Oppositional Defiant Problems were positively associated with Executive Emotional and Behavioral Regulation competences. On the other hand, Rule-Breaking Behaviors and Conduct Problems were negatively associated with Affective Empathy and with Behavioral skills. Our study provides an additional contribution for a better understanding of the complex relationship between empathic competence and executive functions, showing that executive functioning and empathic attitudes interact with each other to regulate aggressive behaviors. This study further corroborates developmental models of empathy and their clinical implications, for which externalizing behaviors could be attenuated by enhancing executive functioning skills.

## 1. Introduction

Feeling empathy for someone means understanding his/her emotions and/or personally experiencing the same; it means creating a customized space in one’s own inner world to host the world of the other. In other words, it refers to the ability to share and comprehend another person’s thoughts and moods [1]. Feeling and understanding the emotions of others are important assumptions to guide one’s actions in a prosocial sense and, particularly, to avoid those behaviors that can cause harm and suffering to the other. The cognitive facet of empathy implies the ability to understand the inner situation of the other and to take his/her own perspective [2]. On the other hand, the affective component of empathy is defined as the ability to share the emotional state of others [3]. The latter implies the involvement of limbic and paralimbic structures and develops earlier than the cognitive one, which assumes a fine-tuned maturation of prefrontal and temporal networks [4].

In light of the close association of empathy with contextual factors, early childhood experiences and social behaviors, it is direct to assume that psychopathological conditions are often convoyed by empathy deficits [5,6]. Empathy deficits have been implicated in several neurodevelopmental disorders, among which autism spectrum disorder (ASD) is the most studied [7,8,9]. ASD have been primarily associated with cognitive empathy deficits [9,10] but the potential role of affective empathy in this framework has been questioned [11].

The reduction or absence of empathy represents the cornerstone of Conduct Disorders (CD) characterized by disruptive and antisocial behaviors [12]. Adults with psychopathic traits show a selective deficit of the affective component of empathy related to impaired emotional responses to facial expressions of feelings of sadness and fear [13,14,15], which is likely due to dysfunctional neuronal circuits underlying the amygdala [16]. A recent study by our group [17] confirmed these findings in a cohort of young boys with CD, corroborating the association between callous-unemotional (CU) traits and affective empathic attitudes. Interestingly, studies aimed at outlining the differences between children with CD and CU traits and ASD [18] show that, while psychopathy seems to be best characterized by a preserved understanding of what the other thinks, with a deficient capacity to share compassionate feelings towards the others, ASD specifically lacks the ability to take others’ perspective. Conversely, a study conducted by Mazza et al., 2014 [11] on a group of adolescents with ASD reported a difficulty in cognitive empathy and a deficit in affective empathy specific for the negative emotional valence, assuming, also for these subjects, the existence of an atypical function and structure of the amygdala [19].

Some evidence has also shown that empathy is compromised in a proportion of children with Attention Deficit and Hyperactivity Disorder (ADHD); in particular, lower levels of social perspective taking are observed [20,21,22]. Indeed, young people with ADHD may have low cognitive empathic attitudes, as demonstrated for instance by the frequently observed unawareness of other children playing the same game [23]. Further corroborating evidence appeared in a recent study by Maoz et al., 2019 [24], who confirmed the existence of a global deficit in both components of empathy by using the Interpersonal Reactivity Index [25] in its self-report form. In another study conducted by the same research group [26], differences in the empathic profile are identified between the Combined and the Inattentive subtypes of ADHD, identifying a greater impairment in the former for all scales of the IRI questionnaire. According to Uekermann et al., 2010 [27], these deficits might be explained at least in part by the impulsive response modalities typically found in ADHD patients, and thus may be linked to dysfunctions of the fronto-striatal brain networks, functionally related to empathic processing and executive functioning. Interestingly, Barkley, 2006 [28], argues that behavioral inhibition deficits, the core symptoms of ADHD children, might impair social cognition skills, but how much they could affect empathic abilities still remains an unsolved question.

Despite the identification of selective or global impairments of empathy in clinical settings, functional studies, aimed at assessing brain correlates of the experience of feeling the emotions of others, found that empathic attitudes are activated through an emotional processing which is regulated both by bottom-up and top-down circuitry within the prefrontal and limbic cortex [29]. An early influential theory, the Perception-Action Model by Preston and de Waal, 2002 [30], based on an evolutionary perspective, proposed that empathy is considered an uncontrolled response that operates automatically and develops early in life. More recently, the Russian Doll Model by the same authors [31] questioned this simplistic view and posited that bottom-up routes of empathic processing and top-down executive modulators are two interrelated systems that develop sequentially. This model has included executive functions as a regulating factor and considered it as a fundamental ground for the development of cognitive empathy [31,32,33].

According to the Russian Doll Model, several components of the empathic response, which have been added layer by layer during evolution, remain functionally integrated [34]. At its core is the perception-action mechanism, which induces a similar emotional state in the observer as in the target. Indeed, its basic expressions are motor mimicry and emotional contagion, representing the functional reactions of newborns and infants according to Hoffman’s developmental theory of empathy [35]. On the other hand, the external layers of the doll, such as empathic concern and perspective taking, are grounded on the previously described socio-affective basis but require a fine-tuned regulation of emotional responses and a distinct perception between self and cognition. Although the outer layers of the doll depend on prefrontal circuitry, they remain functionally connected to the basic perception-action mechanism [31]. Therefore, cognitive empathic attitudes are supported by the neural regions that underlie working memory, executive functioning, emotional regulation and visuo-spatial processing, overpowering the affective representations of the other in a top-to-bottom fashion [31,36]. In other words, the effective control of empathic responses is thus obtained through the executive functioning regulation system, which allows a fine adaptation and modulation of the sharing experience.

A stepwise transition from immature forms of emotional contagion to more sophisticated expressions of prosocial attitudes, as brilliantly theorized by Hoffman’s developmental theory of empathy [37], may be indeed paralleled by the progressive maturation of prefrontal circuitry and executive functions (EF) required to perform a fine-tuned control of such responses. Interestingly, a discrete amount of studies, conducted in community and clinical samples, has repeatedly shown that EF as a whole can modulate empathic attitudes, or in other words people with higher EF competences may better regulate their emotions and reduce perceived distress during the empathic processes [38,39,40,41,42,43,44,45,46]. Moreover, Gökçen et al. [43,44] found positive correlations between EF and empathic attitudes in individuals with ASD traits, suggesting the role of EF in regulating empathic competences in neurodevelopmental disorders. Similarly, a recent study in ADHD patients versus healthy controls by Abdel-Hamid et al., 2019 [47], found significant positive correlations between theory of mind (ToM) and empathy competences and EF performances in the ADHD group but not in the control group. In a recent review, which analyzed fifteen studies conducted on ADHD samples, with or without comorbidities and included mostly male children, Pineda-Alhucema et al., 2018 [48], found the EF most correlated with ToM were inhibitory control, working memory, cognitive flexibility and attention (for further updated details on the relationship between EF and ToM please refer to Andreou et al. 2020 [49]).

It should be noted, however, that in these studies, EF were variably measured through different neuropsychological tasks, such as the Go/No-Go test and the Wisconsin Card Sorting Test, which assess specific EF such as Cognitive Flexibility and Working Memory in laboratory settings. No study, however, used caregiver-reported measures of behavioral patterns for children and adolescents to evaluate EF in everyday life environments. Given the centrality of EFs in controlling behaviors in everyday life, relying only on laboratory measures of EF performances, detected with clinical tests, may limit the confidence and completeness of the clinical evaluation. The measures based on performances, in fact, depict only limited aspects of the EF system in a narrow time frame and do not fully capture the integrated multidimensional decision-making process based on an analysis of the priority which is often that of real life situations [50].

A recent meta-analysis [29] summarized these results, corroborating the evidence that empathy is strongly related to EF, and interestingly its cognitive facet is more closely related to executive skills than the affective one. Particularly, strong relationships were found between cognitive empathy and specific subcomponents of EF, including Inhibitory Control, Working Memory and Cognitive Flexibility, while affective empathy would only correlate to Inhibitory Control. Despite this, it should be observed that this meta-analysis did not consider further subdivisions of EF in their subgroup analyses, such as emotional regulation abilities and several other metacognitive skills. Moreover, no significant effect of age was demonstrated, though a considerable heterogeneity in the age range of samples of included studies is noticeable. Another potential source of bias was the inclusion of the results of three unpublished dissertations. Finally, many of these studies are made up of heterogeneous samples, while the meta-analysis does not take into account psychiatric comorbidities. The authors emphasized indeed that this field of research is still under open investigation, since the results of single studies are somewhat inconsistent and inadequate to draw definite conclusions.

The present study aims to explore possible relationships between the different facets of empathy and the specific subcomponents of EF in a clinical sample of children and adolescents primarily diagnosed with ADHD, compared to children with comorbid ASD or ODD/CD or both. EF profiles have been evaluated through the Behavior Rating Inventory of Executive Function (BRIEF) [51] questionnaire, which provides a structured assessment of EF behaviors in everyday life environments. Since the EF deficits can strongly affect the cognitive and behavioral manifestations of ADHD and ASD [52,53], our aim was indeed to investigate if and how EF are associated with the dimensions of empathy within these neurodevelopmental disorders.

Moreover, the current study examined the role of empathic dimensions and executive skills in regulating the externalizing behaviors typical of some clinical manifestations of ADHD and ODD/CD, such as for instance aggression, oppositional behaviors and rule transgression. It is believed, in fact, that emotional regulation plays an important role in inhibiting aggressive behaviors by implementing perspective-taking abilities and empathic concern towards the others [54].

## 2. Methods

### 2.1. Participants and Diagnostic Procedures

Our study included 151 drug-naïve participants (137 boys, age range 6–18 years old, mean age 9.51 ± 2.64 years) recruited in our third-level Department of Child and Adolescent Psychiatry from March 2019 to December 2019. Subjects underwent a multi-dimensional assessment, through individual clinical evaluations and observations of social interactions within a group of peers, in order to thoroughly investigate ASD symptomatology. The diagnoses were made according to the Diagnostic and Statistical Manual of Mental Disorders–Fifth edition (DSM−5) [55], based on medical history, clinical observations, a structured interview, the Kiddie Schedule for Affective Disorders and Schizophrenia–Present and Lifetime version (K-SADS-PL) [56], and clinical questionnaires, namely the Child Behavior Checklist and the Social Communication Questionnaire, commonly used to assist the diagnostic process.

The Child Behavior Checklist for ages 6 to 18 years (CBCL–6/18) [57,58] is a 118-item scale, completed by parents, with 8 different syndromes scales, a Total Problem Score and two broad-band scores designated as Internalizing Problems and Externalizing Problems. The reliability coefficients (Cronbach’s alpha) of the original validation study were 0.82, 0.81 and 0.82, respectively. The Social Communication Questionnaire (SCQ) [59] is a widely used screening measure for ASD. It was designed as a questionnaire version of the Autism Diagnostic Interview–Revised (ADI-R) [60], the gold standard developmental history measure that is widely used in research and often in clinical practice. Caregivers can rate the individual’s lifetime and/or current characteristics. Compared to other rating scales, the development research was significantly more robust, including good diagnostic validation on participants, and it has been widely adopted by both the research and clinical community worldwide.

Patients were included if they received a diagnosis of ADHD, with or without comorbid psychiatric conditions, including ODD/CD and ASD. The latter was suspected based on either medical history and clinical observations or a total SCQ score above the cut-off value and later confirmed through the administration of the Autism Diagnostic Observation Schedule–Second Edition (ADOS-2) [61]. The exclusion criteria were as follows: presence of comorbid intellectual disability, as detected through formal psychometric assessment by means of the WISC-IV, i.e., when either the Full-Scale Intelligence Quotient or the General Ability Index were below than 70 points; younger than 6 years old or older than 18 years old; current or previous use of psychoactive medications; neurologic impairments or neurodegenerative conditions.

We identified four clinical groups in our samples: “pure” ADHD group (namely, without comorbid ASD and/or ODD/CD, here-in-after referred as the ADHD alone group), including 64 subjects (12.5% girls, mean age 10.02 ± 2.49 years); comorbid ADHD + ODD/CD group (here-in-after referred as the ADHD+ODD/CD group), including 43 subjects (9.3% girls, mean age 9.37 ± 2.95 years); comorbid ADHD + ASD group (here-in-after referred as the ADHD+ASD group), including 19 subjects (5.26% girls, mean age 9.58 ± 2.69 years); comorbid ADHD + ASD + ODD/CD group (here-in-after referred as the ADHD+ODD/CD+ASD group), including 25 subjects (4% girls, mean age 8.40 ± 2.24 years). All participants and parents were informed about assessment instruments and participated voluntarily in the study after written informed consent was obtained for assessment procedures from parents of all children. The study conformed to the Declaration of Helsinki and the Regional Ethics Committee for Clinical Trials of Tuscany, Pediatric Ethics Committee section, approved the study (ethical approval code: GENCU/03/2019).

### 2.2. Clinical Assessment

Patients’ clinical profiles were also assessed by means of several questionnaires, either through self or parental reports. Particularly, the Italian versions of the following measures were used: the Behavior Rating Inventory of Executive Functions–Second version (BRIEF-2) [51] administered to parents of all included children for the assessment of EF profiles; the Antisocial Process Screening Device (APSD) [62] administered to parents of all included children for the evaluation of CU traits; the Basic Empathy Scale (BES) [12], administered to parents of all children aged 11 years old or less, and the Interpersonal Reactivity Index (IRI) [25], administered in its self-report version to adolescents aged 12 years old or more, for the assessment of empathic competences.

The Behavior Rating Inventory of Executive Function–Second version (BRIEF-2) [51] is the updated version of the BRIEF questionnaire which provides a structured assessment of executive function behaviors in everyday life environments, allowing the identification of helpful clinical manifestations in different contexts, i.e., home and school. In its parent-report version that was used in the present study, this tool has been validated for 5- to 18-year-old children and adolescents. BRIEF-2 is a multi-dimensional measure and items are nearly equally distributed across nine factors, each referring to specific executive functions: Inhibit, Self-Monitor, Shift, Emotional Control, Initiate, Working Memory, Plan/Organize, Task Monitor, Organization of Materials. Three composite scales are also identifiable, each including at least two factors: Behavioral Regulation Index (BRI) (including Inhibit and Self-Monitor), Emotional Regulation Index (ERI) (Shift and Emotional Control) and Cognitive Regulation Index (CRI) (Initiate, Working Memory, Plan/Organize, Task Monitor, Organization of Materials). A Global Executive Composite (GEC) score is also computed as sum of the three aforementioned composite indexes.

The Antisocial Process Screening Device (APSD) [62] is a 20-item clinician-administered rating scale normed on a community sample of pre-adolescent children. The available version of the APSD was designed to be completed by parents and teachers; the former was used in the present study. Items are rated on a three-point Likert scale and the scale consists of three main dimensions, based on factor analysis: Narcissism, Impulsivity and Callous-Unemotional. There is substantial support for the validity of the APSD for distinguishing sub-groups of antisocial youths with more severe and aggressive behavior, with characteristics similar to adults with psychopathy [63] (Cronbach’s α = 0.86).

The Basic Empathy Scale (BES) [12] is a self- or parent-reported questionnaire, the latter being used in this study, for children and/or adolescents composed of 20 items distributed across two subscales, respectively, referring to the affective and cognitive components of empathy. Both exploratory and confirmatory analyses of the original validation established good internal consistency for each subscale with Cronbach’s α ranging from 0.79 to 0.85. The Interpersonal Reactivity Index (IRI) was originally developed by Davis, 1980 [25], as a self-reported questionnaire for adults and subsequently adapted for adolescents by Litvack-Miller and colleagues [64]; it is composed of 28 items distributed across four subscales, respectively referring to Fantasy and Perspective-Taking (combined into the Cognitive Empathy subscale), Empathic Concern and Personal Distress (combined into the Affective Empathy subscale).

### 2.3. Data Analysis

Statistical analyses were performed by means of MatLab^®^ (MathWorks, Natick, MA, USA) and RStudio^®^ (RStudio Inc., Boston, MA, USA) software. For each clinical variable with continuous distribution, outliers were defined as observations lying outside the range between [first quartile − 1.5 * interquartile range] and [third quartile + 1.5 * interquartile range] and removed. For each BRIEF-2 subscale-related variable, observations were removed whether the corresponding values at either the Infrequency or the Inconsistency scale was higher than 99° percentile of normalized data. As for the BES and the IRI questionnaires, z-scores were computed for each subscales, i.e., the Cognitive and the Affective Empathy scales; thus, z-scores of the two questionnaires were merged together so that a single pair of variables assessing cognitive and affective empathy was available for all subjects irrespectively of the age.

Analyses of Variance (ANOVA) were used to assess significant differences (*p*-value < 0.05) between clinical variables with continuous distribution. A Tukey post-hoc test was used whenever the ANOVA led to a statistically significant result in order to identify significant comparisons between variables. Spearman’s ranks correlation coefficients were estimated to detect significant relationships between rank values of questionnaire variables. Benjamini and Hochberg’s False Discovery Rate (FDR) correction method [65] for multiple comparisons was applied after assessing significant differences at a traditional significance level of 5%.

Finally, four linear regression models were applied to identify statistically significant relationships between selected subscales of the administered questionnaires. Namely, four subscales of the CBCL (Aggressive Behavior [AB], Rule-Breaking Behavior [RBB], Oppositional Defiant Problems [ODP], Conduct Problems [CP]) were included as dependent variables of the models, whilst the three main indexes of the BRIEF (Behavioral, Emotional and Cognitive Regulation Indexes), the z-scores of the two subscales of the merged Empathy Questionnaire (Affective and Cognitive Empathy) and the SCQ total scores were used as independent variables of the models.

## 3. Results

### 3.1. Questionnaires

**Figure 1:**
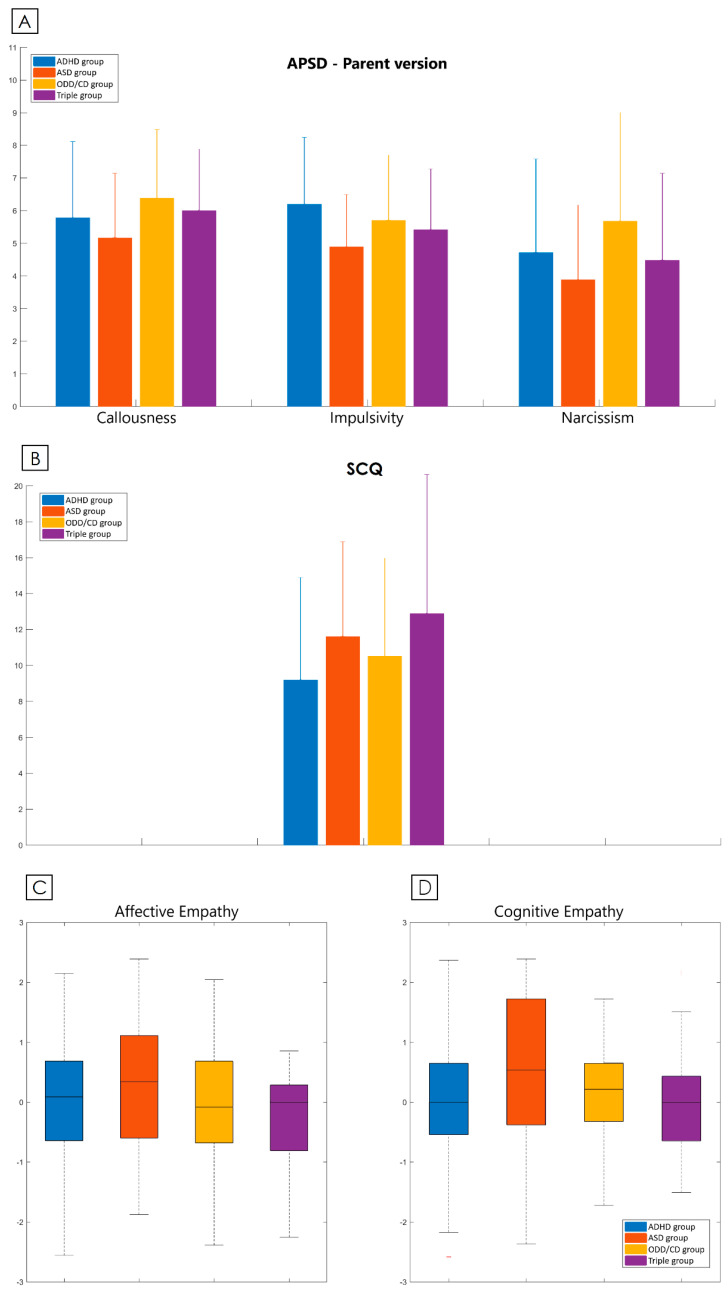
Questionnaires. Scores obtained by the four clinical groups in the Antisocial Process Screening Device (APSD, (**A**)), the Social Communication Questionnaire (SCQ, (**B**)) and the merged Empathy Questionnaire (**C**) are here illustrated. Scores are compared between ADHD group (blue bars), ASD group (red bars), ODD/CD group (yellow bars) and Triple group (purple bars). Graphs represent means with standard deviation bars, except for (**D**) where boxplots represent medians and first and third quartiles with minimum/maximum bars.

Scores obtained by the four clinical groups in the aforementioned questionnaires are reported in Figure 1A–C. No significant differences could be detected in the APSD (Figure 1A) and in the SCQ questionnaires (Figure 1B). Finally, no significant difference emerged neither in the Affective nor in the Cognitive Empathy subscales, neither of the two Empathy questionnaires considered individually (BES and IRI–data not shown) nor in the merged one (Figure 1C).

### 3.2. Correlations

**Figure 2:**
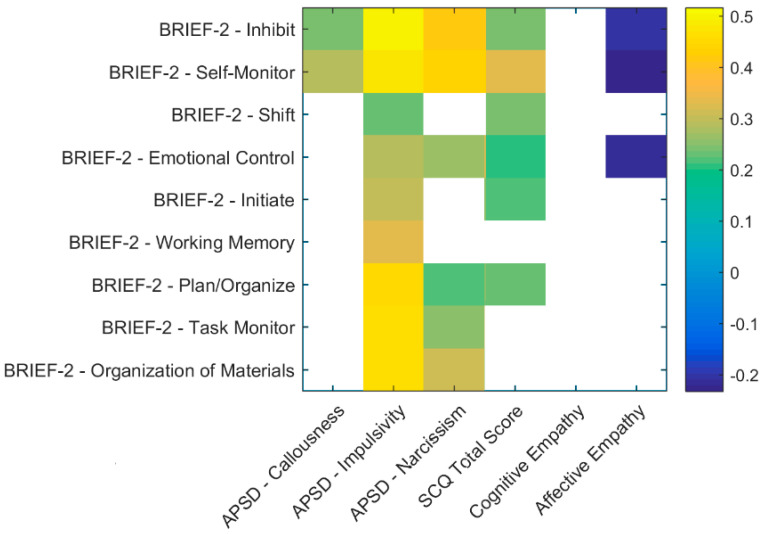
Correlations.

Significant correlations between BRIEF-2-related subscales and APSD, SCQ and Empathy questionnaires are illustrated in Figure 2. Briefly, the Callousness subscale of the APSD was positively associated with the Inhibit and Self-Monitor scales of the BRIEF-2, while the Impulsivity subscale was positively correlated with all scales of the questionnaire. Similar findings were reported for the Narcissism subscale of the APSD, which was positively associated to most subscales of the BRIEF-2 across its three dimensions. The SCQ total score was positively associated to the Emotional and Behavioral Regulation related subscales of the BRIEF-2 and with the Initiate and Plan/Organize subscales. Finally, no significant correlations emerged for the Cognitive Empathy scale, while the Affective Empathy scale was negatively correlated with the Inhibit, the Self-Monitor and the Emotional Control subscales of the BRIEF-2.

Pearson’s linear correlation coefficients were here represented as colored boxes to show only significant relationships between continuous variables of selected questionnaires. The traditional significance level of 5% was corrected by means of Bonferroni’s correction method for multiple comparisons. A color legend bar is displayed on the right.

### 3.3. Regression Models

Finally, a linear regression model was applied to identify statistical relationships between four aggressive/disruptive behavior-related CBCL subscales (AB, RBB, ODP, CP), as dependent variables, and several subscales from the other questionnaires, as independent variables, namely: the three main indexes of the BRIEF-2 (Behavioral, Emotional and Cognitive Regulation Indexes), the two Empathy subscales (Affective and Cognitive Empathy scales) and the SCQ total score.

As displayed in Table 1A–D, a significant positive association was found between both AB (Table 1A) and ODP (Table 1B) subscales of the CBCL, and the Behavioral and Emotional Regulation Indexes of the BRIEF-2. A significant positive relationship emerged, instead, between the RBB (Table 1C) and the CP (Table 1D) subscales of the CBCL, and the Behavioral—but not the Emotional—Regulation Index of the BRIEF-2, while a significant negative relationship was found for the Affective—but not the Cognitive—Empathy subscale.

## 4. Discussion

The first aim of the present study was to explore possible relationships between the different aspects of empathy and specific subcomponents of EF in children and adolescents with ADHD alone, ADHD and ODD/CD and/or ASD. To this aim, our effort was to achieve at least three major objectives: (1) to assess empathic attitudes, CU traits, antisocial behaviors and socio-relational skills in our four groups of patients; (2) to identify potential relationships between these variables and the EF profiles; (3) to explore the interrelated role of the cognitive and affective dimensions of empathy and the EF in regulating antisocial behaviors and aggressiveness. To the best of our knowledge, this is the first study of the kind performed in such a multi-structured sample of children and adolescents.

First, none of the questionnaires used in our study was individually able to discriminate between the four clinical groups. The SCQ is a clinical checklist, based on parents or caregivers report, aimed at identifying the presence of abnormal social and communicative behaviors. Although the SCQ is used as a screening tool for ASD symptoms, the lack of significant differences in our sample between ADHD patients with or without ASD suggests that the social functioning deficits reported in ADHD patients [21], at least in their parents’ judgement, were severe enough to mitigate the difference among groups. It is also likely, however, that, since parents of ADHD children, with or without ASD, are usually more aware of, and worried about, the behavioral consequences of the disorder, socio-communication difficulties could go unnoticed even in patients with a confirmed diagnosis of ASD. A complementary but not alternative explanation may be that our high-functioning ASD patients did not exhibit such a great severity of social and communicative symptoms [66,67], so that caregivers could not be fully aware of their functional consequences.

Similarly, no significant differences emerged between groups in the two questionnaires assessing CU traits. Our finding may suggest that these traits may be trans-nosographic and thus be present not exclusively in ODD/CD, but also in ADHD [68] and in ASD patients [69]. As for the Empathy questionnaires, neither the BES and IRI nor the merged version of the two, did show any significant differences between the four clinical groups. Unfortunately, few data are available in the literature on the empathic attitudes of ADHD children with comorbid psychiatric conditions [70]. One would expect, based on previous findings on non-comorbid conditions, a greater impairment of the affective component for subjects with ADHD in comorbidity with behavioral disorders [17], while a greater impairment of the cognitive component in subjects with ADHD+ASD [10,18]. Our results could be interpreted in light of the subtle complexity of empathy deficits in these neurodevelopmental disorders, which limited the likelihood to find significant differences among comorbid conditions. It should be also taken into account that all our subjects were diagnosed with ADHD and this shared clinical condition may have obscured possible differences among groups. However, this finding further supports the notion that comorbid conditions are not the simple summation of two different disorders. In light of this, ADHD + ASD patients are not simply ASD patients with an additional ADHD, but a specific phenotype, possibly with a lesser cognitive empathic deficit or greater affective empathic impairment. Similarly, ADHD + ODD/CD patients may present a lesser affective empathic deficit or greater cognitive empathic impairment. This hypothesis should be tested by comparing “pure” ASD and ODD/CD with patients with ADHD and comorbidity conditions. Interestingly, recent studies shed some light on future research about specific empathy impairments in ASD and ODD/CD individuals, suggesting that different mechanisms and factors may be involved in empathic problems in such conditions [71,72].

The principal goal of the present study was to assess the reciprocal relationship between empathic attitudes and EF in ADHD patients. A recent meta-analysis [29], mainly including studies performed on healthy subjects, found positive correlations between empathic competences and EF. Gökçen et al. [43,44] reported similar findings in individuals with ASD traits, suggesting the role of EF in regulating empathic competences in neurodevelopmental disorders. Similarly, a more recent study by Abdel-Hamid et al., 2019 [47], identified in ADHD patients, but not in healthy controls, overlapping significant correlations between theory of mind and empathy measures and EF performances at the Trail Making Test, which specifically assesses Cognitive Flexibility and Working Memory skills. As for the so-called “hot” EF, that is the process underlying the affective modulation of behavioral responses [73,74], Miranda et al., 2017 [75], observed significant correlations between social cognition deficits, assessed by means of a specific subscale of the NEPSY–II test [76], and the BRIEF inhibition and emotional control scales in an ADHD sample, while the former are linked to metacognitive deficits in high functioning ASD patients.

In our sample, affective empathic competences, assessed through the BES and the IRI questionnaires, are negatively correlated with Emotional and Behavioral Regulation impairments, identified through the BRIEF-2 questionnaire. The greater the difficulties in “hot” EF, the lower the empathic attitudes, or, in other words, individuals with severe deficits in the EF profile exhibited lower scores on Empathy questionnaires. Nonetheless, no significant correlations were found neither for EF metacognitive domains nor with the cognitive empathy subscale.

Our results suggest that inhibitory and emotional control play an important role in regulating externalizing behavior, even controlling for empathic competences. As stated above, empathic attitudes are activated through an emotional processing which is regulated both by bottom-up and top-down circuitry within the prefrontal and limbic cortex [29,77]. This effective control is achieved through EF modulation, allowing for a fine adjustment of the sharing experience [33]. It should be noted that bottom-up and top-down processes are hardly separable when examined on a behavioral level. Nonetheless, the Russian Doll Model by Preston and de Waal [31] recently posited that top-down executive regulation of empathic processing develops later than bottom-up routes, which are responsible for uncontrolled empathic responses that operates automatically. This model is in line with the Hoffman’s developmental theory of empathy [37], according to which Emotional Contagion would develop earlier than more regulated forms of empathic attitude towards the others.

Our results indicate that EF are more strongly related to the affective empathy than to the cognitive one, which is in disagreement with the results of the aforementioned meta-analysis [29]. Nonetheless, it should be emphasized that the studies of this meta-analysis did not include ADHD patients, who could exhibit such a severe impairment in their EF and a deficit in their cognitive empathic competences, that reciprocal associations would not result in statistical significance. In other words, we posit that ADHD patients are somewhat constrained by their executive dysfunction in an underdevelopment of their empathic attitude, which would be limited to the expression of an emotional contagion from the other.

Furthermore, significant positive relationships were found between several variables of the APSD questionnaire and the BRIEF-2 subscales related to the Emotional and Behavioral Regulation domains. Namely, the higher the scores in CU traits-related questionnaires, the higher the impairment in the Emotional-Behavioral Regulation competences. This finding is in line with a recent study assessing the relationship between CU traits and parent ratings of EF [78]. In particular, this study [78] highlights how CU traits are related to emotional self-regulation, but not to the EF performance scales. Since parental ratings are believed to capture EF behavioral representations, these clinical ratings may be more closely associated with behavioral representations of CU traits, which are also identified by the parents’ report [78].

Finally, in our research, we tried to investigate, in a clinical sample of ADHD patients with psychiatric comorbidities, the relationships between two of the fundamental psychological grounds in the neuropsychologic developmental milestones of children and adolescents, namely EF and empathic attitudes, and how they reciprocally interact to regulate behavioral self-regulation and aggressiveness. Our results confirm a strong and finely structured relationship between these variables, being aggressive behaviors and related disturbances significantly influenced by these underlying processes. Our work highlights two different interactions between EF and empathy to regulate social behaviors in ADHD, where a dysfunction of these elements is essential for aggressive and antisocial behaviors to be carried out towards the others.

A first model identifies aggressiveness and oppositional problems, as indexed through the CBCL questionnaire, mainly associated with difficulties in executive emotional-behavioral regulation processes (such as impulse control and the ability to appropriately regulate one’s behavior according to the context), but not with dimensions of empathy. Interestingly, reactive aggressiveness usually emerges as an impulsive response to hostile-perceived environmental events, often precipitated by irritability and tantrums [79]. This type of aggressiveness has been related to an orbito-frontal cortical dysfunction, for its primary role in adapting system reactivity to stress events [9,17,80].

On the other hand, rule-breaking behaviors and conduct disorders, as indexed through the CBCL questionnaire, likely relate to a proactive type of aggressiveness, which is associated to the activation of self-oriented behaviors to take advantage for personal purposes to the achievement of benefits at the emotional expense of the others’ perspective [79]. Our study confirms that these aspects possibly relate to low levels of affective, but not cognitive, empathy and to impaired behavioral, but not emotional, regulation functioning. It has been hypothesized that proactive aggressiveness might be caused by dysfunctional mechanisms of violence inhibition, which are usually activated by others’ discomfort signs, such as fear and sadness [15]. A deficient activation of this self-control mechanism is usually attributed to abnormal responses of the limbic system, particularly of the amygdala, which have been linked to antisocial behaviors in psychopathy [14,15].

Our results are thus in line with previous studies and further elucidate the complex and intriguing relationships between empathic attitudes and EF. In other words, it seems that both impaired behavioral self-regulation and difficulties in the emotional sharing of others’ internal state may lead to a down-regulation of proactive aggressiveness inhibition systems, while emotional and behavioral regulation functioning systems are essential in preventing more reactive forms of aggressiveness towards the others. Thus, the multifaceted interactions of both “hot” EF and empathic attitudes have a central role in regulating prosocial behaviors.

Our study displays, however, a number of limitations that might undermine the robustness of our conclusions; notably, a marked discrepancy in group size, particularly between the “pure” ADHD and ADHD+ODD/CD+ASD groups, and the absence of a control group of healthy children. The purpose of this preliminary study was, indeed, to explore the issues addressed above; therefore, further studies on larger samples, possibly including heathy controls, a greater number of girls and ASD patients, and children with limited prosocial emotions, will be performed to confirm the results.

## 5. Conclusions

In conclusion, our study provides a further contribution for a better understanding of the complex and intriguing relationship between empathic competence and executive skills. These evidences could be beneficial for the definition of treatment strategies aimed at attenuating externalizing behaviors. Aggressive behaviors would, indeed, be modified by an empathic attitude-oriented approach, which should focus on the underlying executive dysfunction. To sum up, we showed that executive functioning and empathic attitudes interact with each other to regulate aggressive behaviors, being the former more related to reactive aggressiveness and the latter to proactive aggressiveness.

## Figures and Tables

**Table 1:** Linear Regression Models.

*A. CBCL-AB*	Estimates	β-Coefficients	Standard Errors	*p*-Values
*Intercept*	26.3134	0.0000	4.9028	4.21 × 10^−7^ ***
BRIEF-2-BRI	0.7645	0.3891	0.1843	6.49 × 10^−5^ ***
BRIEF-2-CRI	0.0667	0.2932	0.0715	0.3525
BRIEF-2-ERI	0.4610	0.0753	0.1481	0.0023 **
Affective Empathy	−1.2782	−0.1137	0.9909	0.1996
Cognitive Empathy	0.6295	0.0580	0.9616	0.5139
SCQ Score	−0.0541	−0.0306	0.1317	0.6816
***B. CBCL-ODP***	**Estimates**	**β-Coefficients**	**Standard Errors**	***p*** **-** **Values**
*Intercept*	35.6661	0.0000	3.8429	1.23 × 10^−15^ ***
BRIEF-2-BRI	0.5471	0.3891	0.1445	0.0002 ***
BRIEF-2-CRI	0.0535	0.2932	0.0560	0.3420
BRIEF-2-ERI	0.3042	0.0753	0.1161	0.0099 **
Affective Empathy	−0.9299	−0.1137	0.7767	0.2336
Cognitive Empathy	0.1635	0.0580	0.7537	0.8285
SCQ Score	0.0105	−0.0306	0.1032	0.9186
***C. CBCL-RBB***	**Estimates**	**β-Coefficients**	**Standard Errors**	***p*** **-** **Values**
*Intercept*	38.4738	0.0000	3.6169	<2 × 10^−16^ ***
BRIEF-2-BRI	0.6978	0.3891	0.1355	1.11 × 10^−6^ ***
BRIEF-2-CRI	0.0355	0.2932	0.0526	0.5008
BRIEF-2-ERI	0.0637	0.0753	0.1091	0.5606
Affective Empathy	−1.5698	−0.1137	0.7326	0.0343 *
Cognitive Empathy	−0.0574	0.0580	0.7121	0.9359
SCQ Score	−0.0296	−0.0306	0.0968	0.7599
***D. CBCL-CP***	**Estimates**	**β-Coefficients**	**Standard Errors**	***p*** **-** **Values**
*Intercept*	37.5833	0.0000	3.7916	<2 × 10^−16^ ***
BRIEF-2-BRI	0.7870	0.3891	0.1424	2.12 × 10^−7^ ***
BRIEF-2-CRI	−0.0062	0.2932	0.0547	0.9096
BRIEF-2-ERI	0.1749	0.0753	0.1165	0.1359
Affective Empathy	−1.7449	−0.1137	0.7665	0.0247 *
Cognitive Empathy	−0.3663	0.0580	0.7409	0.6220
SCQ Score	−0.1235	−0.0306	0.1008	0.2229

Estimates, standard errors and *p*-values are here presented for four linear regression models between selected subscales of the administered questionnaires. Four subscales of the Child Behavior Checklist questionnaire, namely Aggressive Behaviors (**A**), Oppositional-Defiant Problems (**B**), Rule-Breaking Behaviors (**C**), and Conduct Problems (**D**), were included as dependent variables of the models. The three main indexes of the Behavior Rating Inventory of Executive Functions-Second version (Behavioral, Emotional and Cognitive Regulation Indexes), the two subscales of the Empathy questionnaire (Affective and Cognitive Empathy), and the Social Communication Questionnaire total scores were used as independent variables of the model. * *p*-values < 0.05, ** *p*-values < 0.01, *** *p*-values < 0.001.
